# Supplementary material for: Perturbational phenotyping of human blood cells reveals genetically determined latent traits associated with subsets of common diseases
Source: Nat Genet. 2023 Dec 4;56(1):37–50. doi: 10.1038/s41588-023-01600-x (PMC10786715; doi:10.1038/s41588-023-01600-x)
Supplement: Supplementary file 1 — Supplementary Notes, Supplementary Figs. 1–7 and Supplementary Tables 1–6. [file 41588_2023_1600_MOESM1_ESM.pdf]

nature genetics

Article

https://doi.org/10.1038/s41588-023-01600-x

Perturbational phenotyping of human blood cells reveals genetically determined latent traits associated with subsets of common diseases

In the format provided by the authors and unedited

Supplementary Notes

Estimating the number of independent traits.

While this approach generated high-dimensional blood profiles across all conditions, we also observed highly correlated traits across cell types, readouts, and conditions. We estimated a lower bound for the number of independent traits to be 350 traits based on the number of PCA components required to explain 90% of the variance observed across our screening cohort (**Supplementary Fig. 5**).

Handling of delayed entry in EHR.

We aimed to identify blood readouts associated with the age-of-onset of specific diseases. However, the data in both MGB and UK biobanks only included diagnosis or self-reported disease onset dates, potentially missing early unobserved events. To address this, we set the observation period start as the first date with any available diagnosis in the EHR and used a counting-process style Cox model to handle delayed entry (left truncation)[1].

Differences in PGS associations in MGB Biobank and UKBB.

We observed many significant associations that are unique to either the MGB or UK Biobank (**Supplementary Figs. 6, 7**, **Extended Data File S4, S5**), which is likely a consequence of the differences between these two datasets. When analyzing the MGB cohort, we benefitted from the availability of more precise disease definitions and fine-grained diagnostics/clinical measurements over time, while in the UK Biobank, many of the disease traits were self-reported or mapped to higher-level billing terms to protect privacy. Additionally, the MGB dataset contains many diseases that are not defined in the UK Biobank. Conversely, the significantly larger sample size of the UK Biobank allowed for the identification of many significant disease associations that were not observed in the MGB cohort.

[1] Therneau, T.M., and Grambsch, P.M. (2000). Modeling Survival Data: Extending the Cox Model. Stat. Biol. Heal., 39–77. 10.1007/978-1-4757-3294-8_3

Supplementary Figures

Supplementary Fig. 1 Distribution of blood readouts under baseline and perturbation conditions.

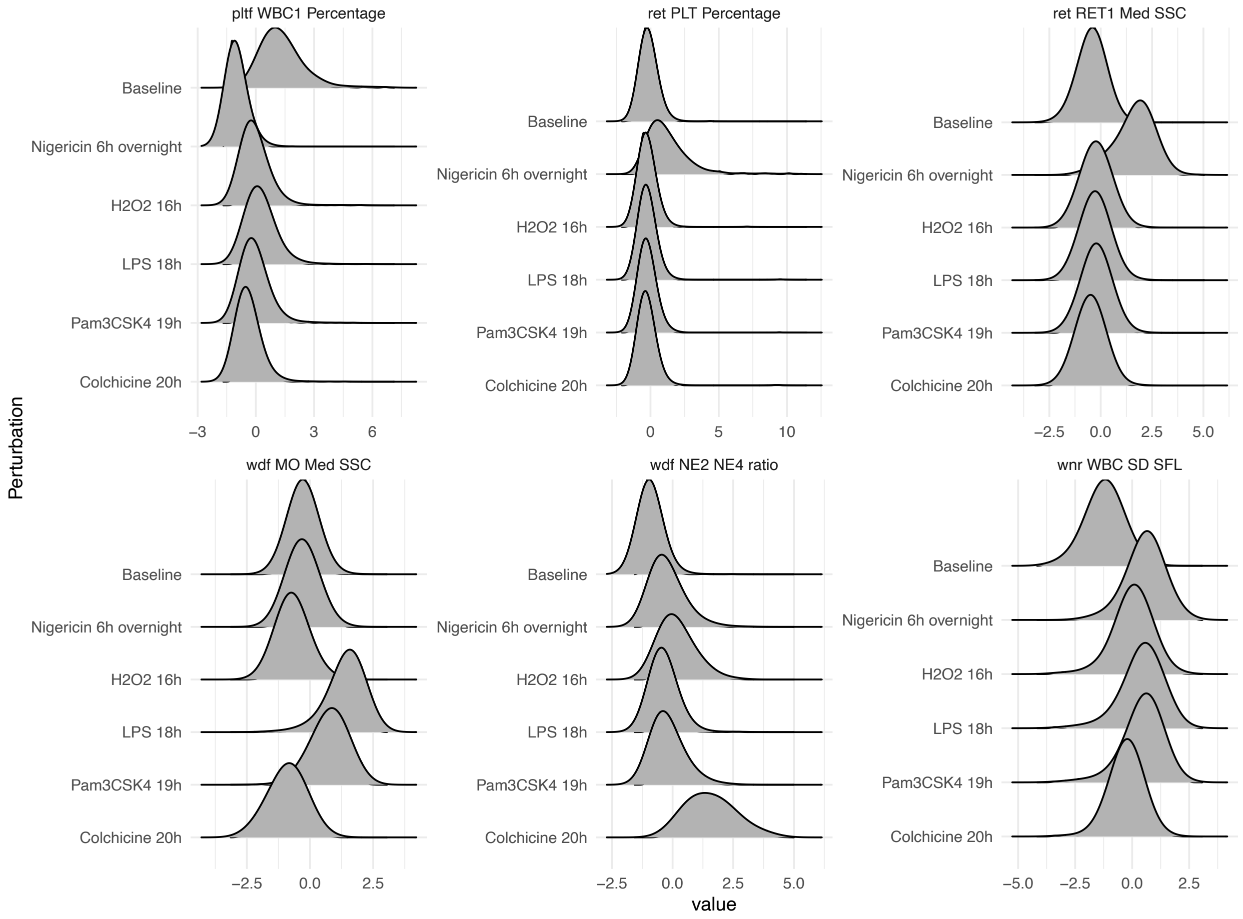

Quantitative parameters were defined by first gating cytometry readouts by cell type and calculating statistical parameters such counts, or median and standard deviation in dimensions such as side scatter (SSC) and side fluorescence (SFL) for each cell type.

Supplementary Fig. 2 Clinical associations of blood readouts with blood-perturbation responses.

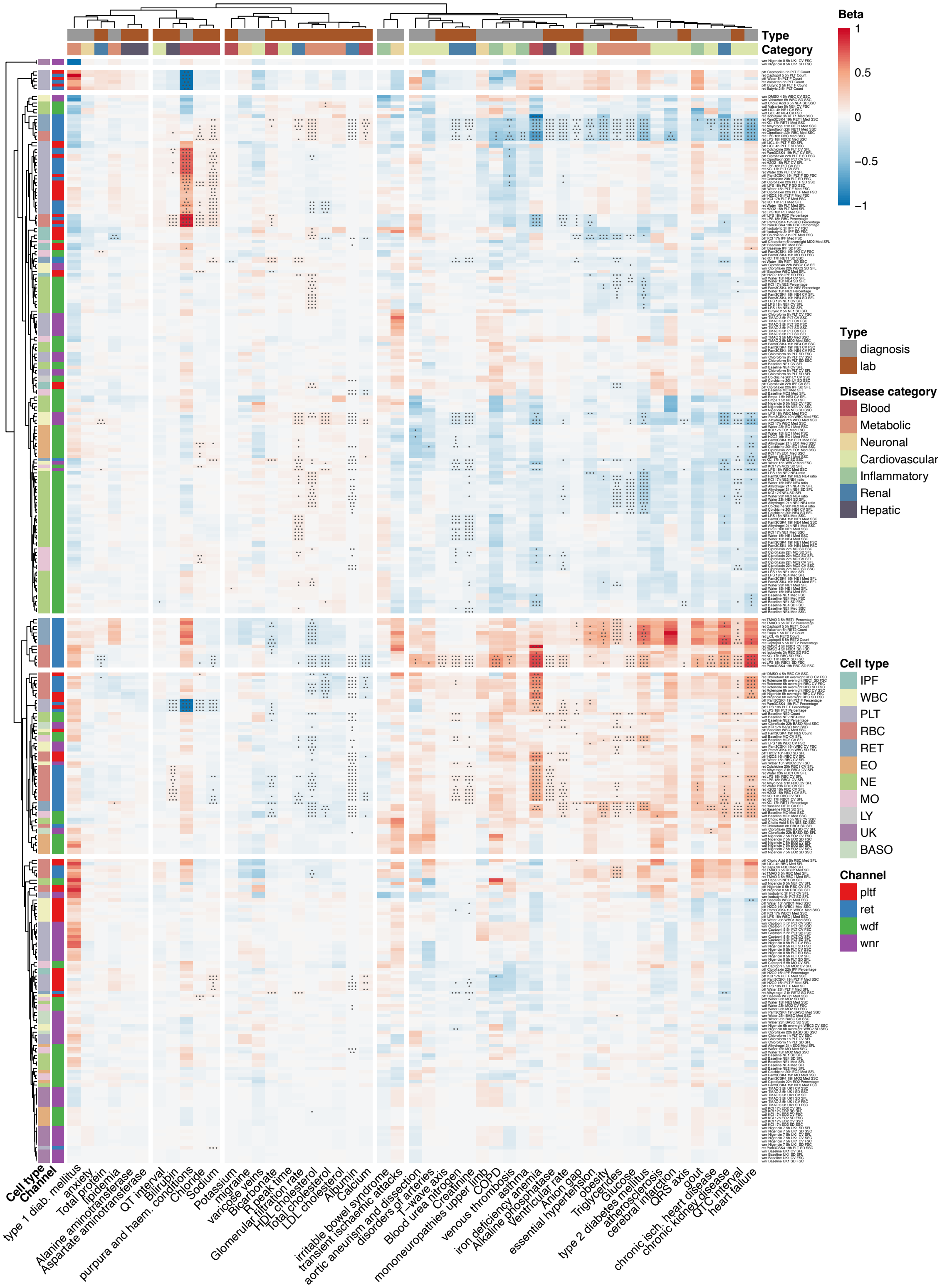

Pairwise association between quantile-transformed blood readouts and clinical lab values or diagnostic codes. Association beta coefficients were estimated using linear and logistic regression models for quantitative lab measurements and binary traits (ICD10 diagnostic codes), respectively. Positive associations are shown in red, negative associations are shown in blue. P values are based on two-sided t-tests and Wald tests. Points indicate significant associations after multiple testing correction using FDR across all tested diseases and blood traits (50 clinical outcomes and 327 blood readouts) with adjusted p-value thresholds: · 0.001, · · 0.001, · · · 0.0001. See Table S3 for clinical trait definitions using diagnostic codes, and **Extended Data File S2** for all association results with FDR < 0.1.

Supplementary Fig. 3 Hazard ratio estimates for time to disease onset computed using perturbation blood-response PGS in meta-analysis of MGB Biobank and UKBB.

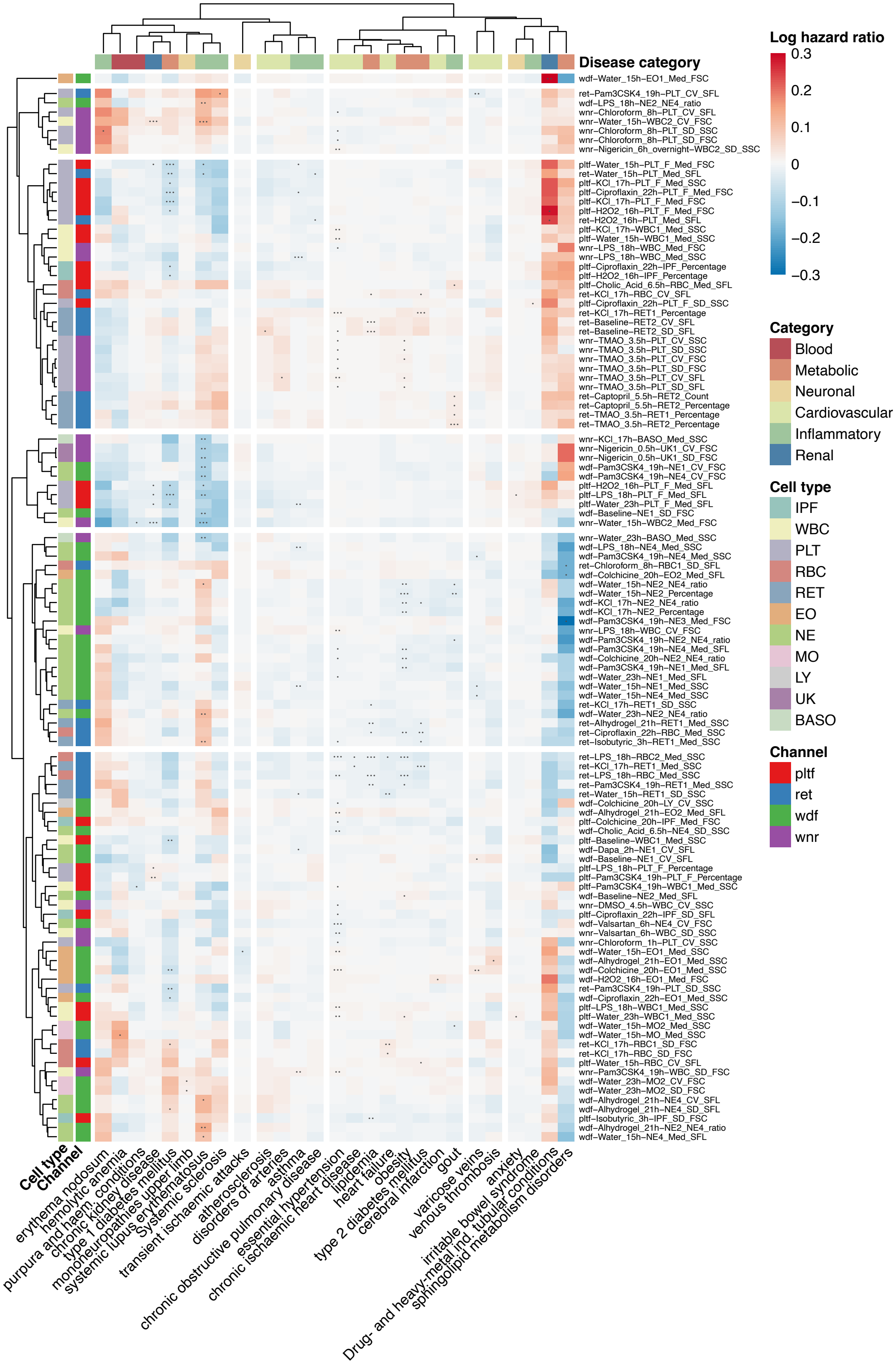

Hazard ratio estimates in time-to-event models from a meta-analysis of clinical outcomes in the MGB Biobank and the UK Biobank. Time to first diagnostic code or diagnosis date in the medical problem list was modeled using sex, the first two genetic principal components, and scaled blood-response polygenic scores in each cohort. Meta-analysis P values are based on two-sided z-scores in random effects models. Points indicate significant associations after multiple testing correction across all tested diseases and blood traits (20 clinical outcomes and 327 blood readouts) with adjusted p-value thresholds: · 0.05, · · 0.01, · · · 0.001. Shown are blood traits and outcomes with at least one significant association.

Supplementary Fig. 4 RBC side fluorescence and covariates.

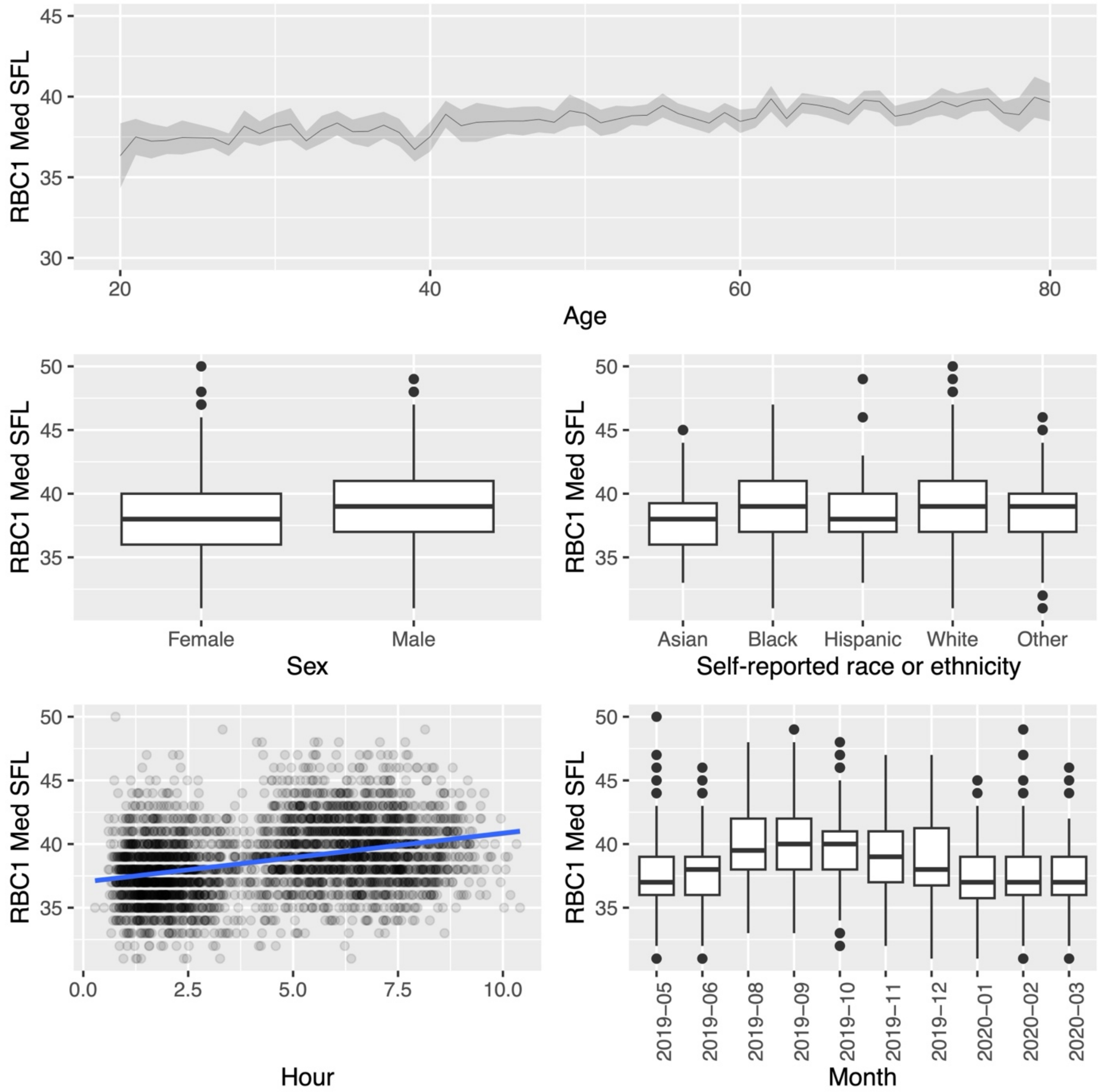

Biological and technical covariates affecting blood readouts under baseline conditions. We observed significant associations with participants age, sex, race, the time between blood draw and analysis as well as the month of the study in multiple blood readouts, RBC1 SFL is shown here as example. These variables together with genotyping-specific covariates (first ten principal components of genotype matrix, genotyping platform, and genotyping batch) were used as covariates in genetic association models. Data shown based on 3391 baseline samples, error bands in age panel show 95%CI. Boxplots represent the interquartile range (IQR) between the first and third quartiles as the box, the median as the line inside the box, and the whiskers extend from the box to the largest and smallest values within 1.5 * IQR, with any points outside of this range shown as individual outliers.

Supplementary Fig. 5 Estimated number of independent traits.

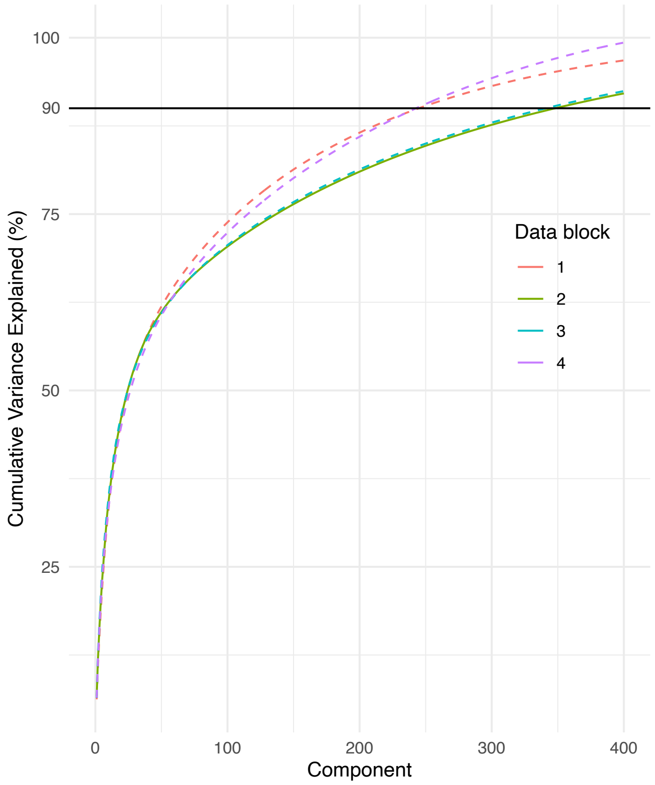

We identified blocks of measurements (subsets of subjects and conditions) with low missingness over the course of the study. We then calculated the number of PCA components that cumulatively explain 90% of the observed variance in these contiguous blocks of blood measurements. We estimate a lower bound of at least 350 independent traits given our current data. A more precise estimation would require complete measurements across all conditions in larger numbers of subjects simultaneously.

Supplementary Fig. 6 Hazard ratio estimates for time to disease onset computed using perturbation blood-response PGS in the MGB Biobank.

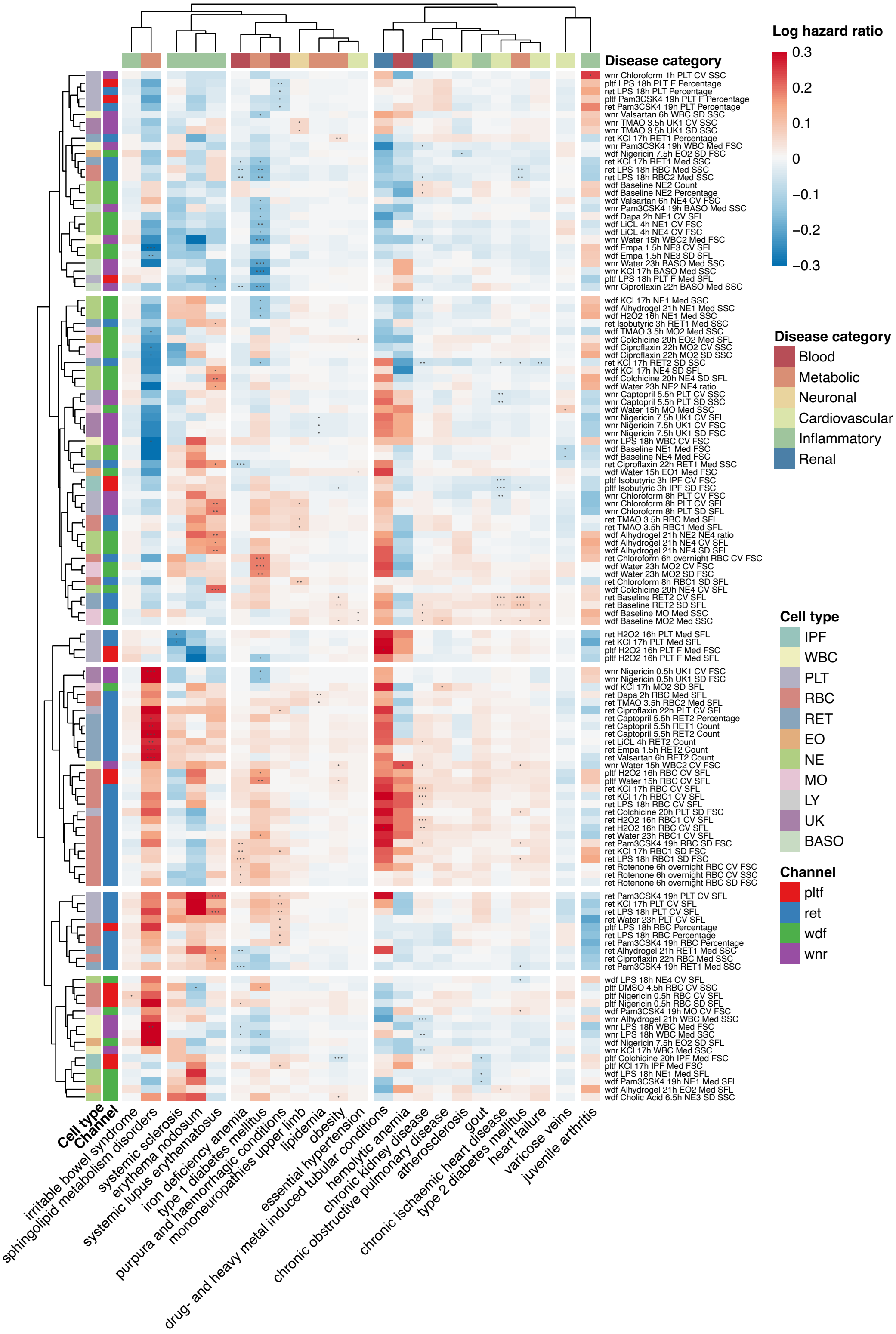

Hazard ratio estimates in time-to-event models for clinical outcomes in the MGB Biobank. Time to first diagnostic code or diagnosis date in the medical problem list was modeled using sex, the first two genetic principal components and scaled blood-response polygenic scores. P values are obtained from Cox PH models with delayed entry (two-sided test) and corrected for multiple testing using FDR. Points indicate significant associations after multiple testing correction across all tested diseases and blood traits (20 clinical outcomes and 327 blood readouts) with adjusted P value thresholds: · 0.05, · · 0.01, · · · 0.001. Shown are blood traits and outcomes with at least one significant association.

Supplementary Fig. 7 Hazard ratio estimates for time to disease onset computed using perturbation blood-response PGS in the UKBB.

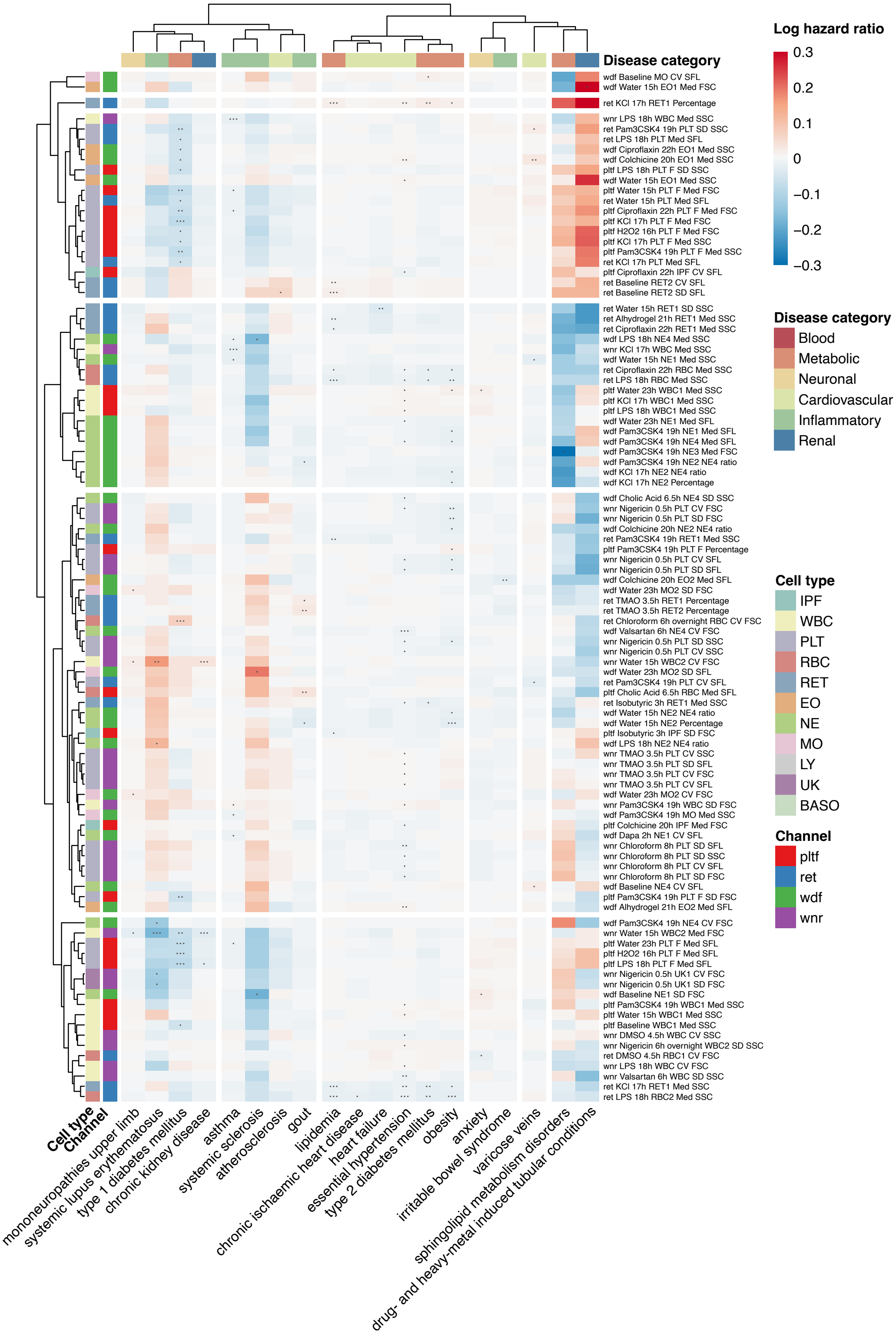

Hazard ratio estimates in time-to-event models for clinical outcomes in the UKBB. Time to first diagnostic code or diagnosis date in the medical problem list was modeled using sex, the first two genetic principal components and scaled blood-response polygenic scores. P values are obtained from Cox PH models with delayed entry (two-sided test) and corrected for multiple testing using FDR. Points indicate significant associations after multiple testing correction across all tested diseases and blood traits (30 clinical outcomes and 327 blood readouts) with adjusted P value thresholds: · 0.05, · · 0.01, · · · 0.001. Shown are blood traits and outcomes with at least one significant association.

Supplementary Tables

Supplementary Table 1. Perturbation conditions.

Condition	Incubation time	Concentration	Blood profiles	Genotypes	QCed EUR Genotypes
Baseline	0h		3223	2271	1705
Water 6h	6h at 39C		717	265	164
LPS 6h	Add LPS, 6h at 39C	1 ug/mL	719	267	167
Chloroform 6h	4h at 39C, add chloroform, 2h at 39C		717	266	168
LPS Nigericin 6h	Add LPS, 4h at 39C, add nigericin, 2h at 39C	1 ug/mL LPS 2.5uM Nigericin	733	270	169
Nigericin 6h	4h at 39C, add nigericin, 2h at 39C	2.5uM	723	264	165
Nigericin 6h overnight	Overnight at 4C, 4h at 39C, add nigericin, 2h at 39C	2.5uM	1482	1276	974
Chloroform 6h overnight	Overnight at 4C, 4h at 39C, add chloroform, 2h at 39C		1472	1275	966
Rotenone 6h overnight	Overnight at 4C, 4h at 39C, add rotenone, 2h at 39C	2.5uM	1458	1255	954
Water 15h	15h at 39C		3303	1972	1429
H2O2 16h	Add H2O2, 16h at 39C	20mM	3258	1940	1404
KCl 17h	Add KCl, 17h at 39C	10mM	3304	1971	1427
LPS 18h	Add LPS, 18h at 39C	1 ug/mL	3294	1970	1434
Pam3CSK4 19h	Add Pam3CSK4, 19h at 39C	0.1 ug/mL	3227	1931	1406
Colchicine 20h	Add colchicine, 20h at 39C	30 ng/mL	3199	1941	1409
Alhydrogel 21h	Add alhydrogel, 21h at 39C	120 ug/mL	3255	1936	1401
Ciproflaxin 22h	Add ciproflaxin, 22h at 39C	100 ug/mL	3231	1944	1407
Water 23h	23h at 39C		3331	1995	1441
Nigericin 0.5h	Add nigericin, 0.5h at 39C	2.5 uM	700	500	390
Chloroform 1h	Add chloroform, 1h at 39C		653	462	361
Empa 1.5h	Add empaglizflozin, 1.5h at 39C	2.14mM	692	498	390
Dapa 2h	Add dapagliflozin, 2h at 39C	2.13mM	641	449	348
Butyric 2.5h	Add butyric acid, 2.5h at 39C	16mM	692	493	385
Isobutyric 3h	Add isobutyric acid, 3h at 39C	16mM	679	484	378
TMAO 3.5h	Add trimethylamine N-oxide, 3.5h at 39C	166mM	671	478	376
LiCl 4h	Add LiCl, 4h at 39C	39.22mM	693	498	390

DMSO 4.5h	Add DMSO, 4.5h at 39C		696	497	390
Water 5h	5h at 39C		694	496	389
Captopril 5.5h	Add captopril, 5.5h at 39C	200mM	678	487	384
Valsartan 6h	Add valsartan, 6h at 39C	428uM	674	492	386
Cholic Acid 6.5h	Add cholic acid, 6.5h at 39C	21uM	674	485	381
Acetic Acid 7h	Add acetic acid, 7h at 39C	12mM	670	485	383
Nigericin 7.5h	Add nigericin, 7.5h at 39C	2.5uM	653	470	368
Chloroform 8h	Add chloroform, 8h at 39C		656	467	366
Nigericin 12 + 6h	4h 39C, add nigericin, 2h at 39C	2.5uM	1238	292	164
Chloroform 12 + 6h	4h 39C, add chloroform, 3h at 39C		1303	301	166
Rotenone 12 + 6h	4h 39C, add rotenone, 4h at 39C	2.5uM	1310	306	167

Supplementary Table 2. Abbreviations used for cell traits.

Abbreviation	Full Name
**Sysmex Dye/Channels**	
WDF	White Cell Differential Channel by Fluorescence Channel
WNR	White Count and Nucleated Red Blood Cells Channel
PLT-F	Platelet-F Channel
RET	Reticulocyte Channel
**Cell Types**	
RBC	Red blood cell
WBC	White blood cell
NE	Neutrophil
MO	Monocyte
BASO	Basophil
EO	Eosinophil
LY	Lymphocyte
IPF	Immature Platelet Fraction
PLT	Platelet
RET	Reticulocyte
UK	Unknown Cell Population
**Statistical Parameters**	
Med	Median
CV	Robust Coefficient of Variation
SD	Robust Standard Deviation
**Measurements**	
FSC	Forward Scatter
SSC	Side Scatter
SFL	Side Fluorescence

Supplementary Table 3. Demographics of screening cohort.

		Missing	Overall
n			4723
Age, mean (SD)		0	54.8 (17.2)
Sex, n (%)	Female	0	2368 (50.1)
	Male		2354 (49.8)
	Unknown		1 (0.0)
Self-reported race or ethnicity, n (%)	Asian	0	177 (3.7)
	Black		437 (9.3)
	Hispanic		76 (1.6)
	Other		347 (7.3)
	White		3686 (78.0)
Tobacco, n (%)	Never	66	2845 (61.1)
	Not Asked		5 (0.1)
	Passive		13 (0.3)
	Quit		1546 (33.2)
	Yes		248 (5.3)
BMI, mean (SD)		144	28.6 (6.6)
Genotyped, n (%)	False	0	2038 (43.2)
	True		2685 (56.8)

Supplementary Table 4. ICD10 diagnostic code definitions used for disease associations.

Category	Name	ICD10 Prefix	UKBB Trait ID
Blood	iron deficiency anemia	D50	f130622
Blood	hemolytic anemia	D59	f130638
Blood	purpura and hemorrhagic conditions	D69	f130658
Metabolic	type 1 diabetes mellitus	E10	f130706
Metabolic	type 2 diabetes mellitus	E11	f130708
Metabolic	obesity	E66	f130792
Metabolic	sphingolipid metabolism disorders	E75	f130808
Metabolic	lipidemia	E78	f130814
Neuronal	anxiety	F41	f130906
Neuronal	transient ischemic attacks	G45	f131056
Neuronal	mononeuropathies upper limb	G56	f131074
Cardiovascular	essential hypertension	I10	f131286
Cardiovascular	chronic ischemic heart disease	I25	f131306
Cardiovascular	heart failure	I50	f131354
Cardiovascular	cerebral infarction	I63	f131366
Cardiovascular	atherosclerosis	I70	f131380
Cardiovascular	aortic aneurism and dissection	I71	f131382
Cardiovascular	disorders of arteries	I77	f131390
Cardiovascular	venous thrombosis	I82	f131400
Cardiovascular	varicose veins	I83	f131402
Inflammatory	chronic obstructive pulmonary disease	J44	f131492
Inflammatory	asthma	J45	f131494
Inflammatory	irritable bowel syndrome	K58	f131638
Inflammatory	erythema nodosum	L52	f131758
Inflammatory	juvenile arthritis	M08	f131854
Inflammatory	gout	M10	f131858
Inflammatory	systemic lupus erythematosus	M32	f131894
Inflammatory	systemic sclerosis	M34	f131898
Renal	drug- and heavy-metal-induced tubulo-interstitial and tubular conditions	N14	f132024
Renal	chronic kidney disease	N18	f132032

Supplementary Table 5. Reagents and assays.

REAGENT	SOURCE	IDENTIFIER
Antibodies		
CD11b Pacific Blue, clone ICRF44	BioLegend	3013215
CD62L Alexa Flour 488, clone DREG-56	BioLegend	304816
Chemicals, peptides, and recombinant proteins		
2-Deoxy-D-Glucose	MedChemExpress	HY-13966
Triacsin C	TOCRIS	2472
Lipopolysaccharides from Escherichia coli	Sigma-Aldrich	L4391
Chloroform	Sigma-Aldrich	C2432
Hydrogen Peroxide	Sigma-Aldrich	216763
Potassium Chloride	Sigma-Aldrich	P9333
Colchicine	Sigma-Aldrich	C9754
Ciprofloxacin	Sigma-Aldrich	17850
Butyric Acid	Sigma-Aldrich	W222119
Trimethylamine N-Oxide	Sigma-Aldrich	317594
Lithium Chloride	Sigma-Aldrich	203637
Captopril	Sigma-Aldrich	C4042
Valsartan	Sigma-Aldrich	PHR1315
Cholic Acid	Sigma-Aldrich	C1129
Acetic Acid	Sigma-Aldrich	695092
Rotenone	Santa Cruz Biotechnology	203242
Pam3CSK4	InvivoGen	tlrl-pms
Alhydrogel adjuvant 2%	InvivoGen	Vac-alu-250
Empagliflozin	Advanced ChemBlocks	G-7261
Dapagliflozin	Millipore Sigma	SML2804
Isobutyric Acid	Millipore Sigma	I1754
Commercial assays		
XPp Real Time ATP Rate assay kit	Agilent	103591-100
EasySep™ Direct Human Neutrophil Isolation Kit	STEMCELL	19666
EasySep™ RBC Depletion Reagent	STEMCELL	18170
AnnexinV Alexa Flour 555	Invitrogen	A35108
SytoX Green Nucleic Acid Stain	Invitrogen	S7020
CellROX Deep Red	Invitrogen	C10422

Supplementary Table 6. Guide RNA targeting sequences.

Gene	gRNA1 target sequence	gRNA2 target sequence
hk1	GACATGGACGAAACGCTTCA	ATCCGTTCTCCATTTCCCGT
pfkpa	GTAGACGCAAGAAGTGCAGA	TCCCAATAACTGTGCCACCC
pfkpb	AGGAGCGCCAACATTCAACA	ATATCCACAATAACCCCCCA
acsl1a	CTCACTTACCGGCACCTCCA	TGCACGTCGCTCTATCCTAA
acsl1b	AGGTACATCACACACCACCG	ACATGGGCAAAACTCACCGG
