# Supplementary material for: Perturbational phenotyping of human blood cells reveals genetically determined latent traits associated with subsets of common diseases
Source: Nat Genet. 2023 Dec 4;56(1):37–50. doi: 10.1038/s41588-023-01600-x (PMC10786715; doi:10.1038/s41588-023-01600-x)
Supplement: Supplementary file 2 — Reporting Summary [file 41588_2023_1600_MOESM2_ESM.pdf]

Corresponding author(s): Max Homilius, Wandi Zhu, Calum A. MacRae, Rahul C. Deo

Last updated by author(s): Oct 20, 2023

Reporting Summary

Nature Portfolio wishes to improve the reproducibility of the work that we publish. This form provides structure for consistency and transparency in reporting. For further information on Nature Portfolio policies, see our Editorial Policies and the Editorial Policy Checklist.

Statistics

For all statistical analyses, confirm that the following items are present in the figure legend, table legend, main text, or Methods section.

n/a	Confirmed	
☐	☒	The exact sample size (*n*) for each experimental group/condition, given as a discrete number and unit of measurement
☐	☒	A statement on whether measurements were taken from distinct samples or whether the same sample was measured repeatedly
☐	☒	The statistical test(s) used AND whether they are one- or two-sided<br>*Only common tests should be described solely by name; describe more complex techniques in the Methods section.*
☐	☒	A description of all covariates tested
☐	☒	A description of any assumptions or corrections, such as tests of normality and adjustment for multiple comparisons
☐	☒	A full description of the statistical parameters including central tendency (e.g. means) or other basic estimates (e.g. regression coefficient) AND variation (e.g. standard deviation) or associated estimates of uncertainty (e.g. confidence intervals)
☐	☒	For null hypothesis testing, the test statistic (e.g. *F*, *t*, *r*) with confidence intervals, effect sizes, degrees of freedom and *P* value noted<br>*Give P values as exact values whenever suitable.*
☒	☐	For Bayesian analysis, information on the choice of priors and Markov chain Monte Carlo settings
☒	☐	For hierarchical and complex designs, identification of the appropriate level for tests and full reporting of outcomes
☒	☐	Estimates of effect sizes (e.g. Cohen's *d*, Pearson's *r*), indicating how they were calculated

Our web collection on statistics for biologists contains articles on many of the points above.

Software and code

Policy information about availability of computer code

Data collection	Study data of the screening cohort were collected using REDCap v13.1
Data analysis	Plink 1.9 and 2.0, Michigan Imputation Server (docker image) 1.5.7, R 4.2, data.table 1.14.6, ggplot2 3.4.1, survival 3.5-3, survminer 0.4.9, circos 0.69-9, fujiplot, qvalue 2.4.2, PCAtools 2.12.0, fastICA 1.2-3, python 3.9, pandas 1.4.1, numpy 1.19.5, statsmodels 0.13.2, FlowJo 10.8.1, GraphPad Prism 9. Main analysis scripts are available at https://github.com/mxhm/blood_perturbation_gwas.

For manuscripts utilizing custom algorithms or software that are central to the research but not yet described in published literature, software must be made available to editors and reviewers. We strongly encourage code deposition in a community repository (e.g. GitHub). See the Nature Portfolio guidelines for submitting code & software for further information.

Data

Policy information about availability of data

All manuscripts must include a data availability statement. This statement should provide the following information, where applicable:

- Accession codes, unique identifiers, or web links for publicly available datasets
- A description of any restrictions on data availability
- For clinical datasets or third party data, please ensure that the statement adheres to our policy

Individual-level data sharing is subject to restrictions imposed by patient consent and local ethics review boards. GWAS summary statistics have been submitted to the GWAS catalog database (study ids: GCST90257015-GCST90257105). PGS are available at https://doi.org/10.6084/m9.figshare.24354235.

Human research participants

Policy information about studies involving human research participants and Sex and Gender in Research.

Reporting on sex and gender	Self-reported sex was used as covariate in clinical association analyses and genetic association analyses of blood readouts, as well as polygenic score analyses.
Population characteristics	Population characteristics are described in Extended Data Table 2.
Recruitment	Subjects were recruited in accordance with IRB 2019P003155 from multiple phlebotomy clinics in the MassGeneralBrigham hospital system. Subjects were recruited at the time of check-in/registration for their clinical blood draw. Once patients consented, the patient underwent their clinical blood draw first, then the phlebotomist drew blood tubes for the research study. Patients were able to request study staff to fully discuss risks, benefits, and obtain consent at the time of the visit. The study inclusion criteria were age>18, already scheduled to have blood drawn as part of routine clinical care, and ability to provide informed consent. There is no selection bias in recruitment.
Ethics oversight	MassGeneralBrigham hospital

Note that full information on the approval of the study protocol must also be provided in the manuscript.

Field-specific reporting

Please select the one below that is the best fit for your research. If you are not sure, read the appropriate sections before making your selection.

☒ Life sciences ☐ Behavioural & social sciences ☐ Ecological, evolutionary & environmental sciences

For a reference copy of the document with all sections, see nature.com/documents/nr-reporting-summary-flat.pdf

Life sciences study design

All studies must disclose on these points even when the disclosure is negative.

Sample size	Samples were collected in multiple phlebotomy clinics over the course of several years. The sample size was not calculated prior to sample collection, as we tried to maximize the number of subjects recruited to maximize power for testing genome-wide associations. We gathered blood samples for 4723 subjects and genotyped 2685 of them (Extended Data Table 2). The number of samples varied for the perturbation conditions from over 641 to 3223 blood samples (Extended Data Table 1).<br>For functional validation studies, sample size calculation was not performed. Sample sizes were chosen based on comparisons to prior similar studies. Notably, for experiments using human blood samples, samples from at least 8 donors were used for each condition/experiment. Although there may be substantial variability among individual donors, the compound treatments used in our study elicited pronounced effects, which were consistently observed with our chosen sample sizes.
Data exclusions	Samples were excluded based on pre-established criteria for phenotypic and genetic quality control (online methods). For clinical associations, subjects with organ transplants were excluded due to the effects of immunosuppressant medications.
Replication	We analyzed all available samples in the genome wide association studies and no additional datasets are available for replication of the perturbation responses. To reduce risk of false-positive results, we performed quality control on phenotypic and genetic data (online methods).<br>For other experiments involving donor blood samples, each experiment was performed on at least two independent days on independent samples. For experiments involving zebrafish, at least two clutches of zebrafish embryos were used for each experiment. All attempts at replication were successful.
Randomization	Randomization is not applicable, since the main study design is a collection of genome-wide association studies of blood cell readouts. The goal of our study is to identify naturally occurring genetic variants associated with measured blood traits within a population, which does not involve assigning participants to different experimental conditions.
Blinding	GWAS analyses were not blinded since linking genotype and phenotype information was necessary for statistical analyses.<br>Studies using zebrafish were blinded. Functional studies using human blood samples and compound treatments were not blinded, however, the data were analyzed with the same flow cytometry gating strategy and the analyses were standardized with FlowJo.

Reporting for specific materials, systems and methods

We require information from authors about some types of materials, experimental systems and methods used in many studies. Here, indicate whether each material, system or method listed is relevant to your study. If you are not sure if a list item applies to your research, read the appropriate section before selecting a response.

Materials & experimental systems

n/a	Involved in the study
☐	☒ Antibodies
☒	☐ Eukaryotic cell lines
☒	☐ Palaeontology and archaeology
☐	☒ Animals and other organisms
☒	☐ Clinical data
☒	☐ Dual use research of concern

Methods

n/a	Involved in the study
☒	☐ ChIP-seq
☐	☒ Flow cytometry
☒	☐ MRI-based neuroimaging

Antibodies

Antibodies used	Pacific Blue anti-human CD11b antibody (Biolegend, Clone ICRF44, catalog number 3013215) and Alexa Fluor 488 anti-human CD62L antibody (Biolegend, Clone DREG-56, catalog number 304816).
Validation	All antibodies used in this study have been validated by the manufacture. They have verified reactivity to human cell/tissue. According to the manufacture, "Each lot of this antibody is quality control tested by immunofluorescent staining with flow cytometric analysis."

Animals and other research organisms

Policy information about studies involving animals; ARRIVE guidelines recommended for reporting animal research, and Sex and Gender in Research

Laboratory animals	zebrafish (danio rerio) larvae (2-4 day post fertilization)
Wild animals	No wild animals were used in the study
Reporting on sex	Zebrafish do not have heteromorphic sex chromosomes and their sex are not determined until older than 20-25 day post fertilization. As we used zebrafish larvae in this study, there is no sexual differentiation at this stage.
Field-collected samples	No field collected samples were used in the study
Ethics oversight	Brigham and Women's Hospital Standing Committee on Animals

Note that full information on the approval of the study protocol must also be provided in the manuscript.

Flow Cytometry

Plots

Confirm that:

☒ The axis labels state the marker and fluorochrome used (e.g. CD4-FITC).

☒ The axis scales are clearly visible. Include numbers along axes only for bottom left plot of group (a 'group' is an analysis of identical markers).

☒ All plots are contour plots with outliers or pseudocolor plots.

☒ A numerical value for number of cells or percentage (with statistics) is provided.

Methodology

Sample preparation	For Sysmex-based measurements, whole blood was collected in 8.5mL ACD tubes (BD 364606). Barcoded sample tubes with patient and perturbation identifiers were aligned and prepared batch-wise, by aliquoting 700ul of whole blood into a grid of 5mL round bottom tubes. All perturbation compounds were added to blood at specified time points and transferred to incubator shakers (39C, 200 RPM). After incubation, tubes were placed in automated sampling racks and profiled using the Sysmex XN-1000.<br>For isolated neutrophil measurements. We used EasySep Direct Human Neutrophil Isolation Kit (#19666, STEMCELL) to isolate neutrophils according to manufacture protocols. Post isolation, neutrophils were resuspended in Tyrode's solution as described previously. To characterize the NE2-like cell population using flow cytometry, neutrophils were isolated from whole blood samples that were incubated at 37°C for 17h, and then labeled with apoptosis indicators, Sytox green (S7020, ThermoFisher Scientific), and R-PE conjugated annexin V (ThermoFisher Scientific). The labeled neutrophils were then subjected to permeabilization using Sysmex WDF Lysercell (Sysmex) and staining with Fluorocell WDF dye (Sysmex). The samples were analyzed 5 minutes post the addition of Flurocell WDF dye.<br>To characterize neutrophil activation and ROS, isolated neutrophils were labeled with Pacific Blue anti-human CD11b antibody (Biolegend, Clone ICRF44) and Alexa Fluor 488 anti-human CD62L antibody (Biolegend, Clone DREG-56). Cells were then subsequentially labeled with CellROX Deep Red Reagent (ThermoFisher Scientific, C10422) at 37°C for 30 minutes. Cells

were washed and resuspended in staining buffer prior to flow cytometry analyses.

Instrument	BD FACSymphony, Sysmex XN-1000
Software	FlowJo v10.8.1
Cell population abundance	Sorting was not employed in this study
Gating strategy	Gates were empirically defined based on densities of measured cells under baseline and treated conditions across all subjects. Gates are shown in Figure 1B.

☒ Tick this box to confirm that a figure exemplifying the gating strategy is provided in the Supplementary Information.
